# Microstructure and Charpy Impact Toughness of a 2.25Cr-1Mo-0.25V Steel Weld Metal

**DOI:** 10.3390/ma13133013

**Published:** 2020-07-06

**Authors:** Kefan Wu, Yingjie Yan, Rui Cao, Xinyu Li, Yong Jiang, Fei Yang, Xingwang Jia, Jianhong Chen

**Affiliations:** 1 State Key Laboratory of Advanced Processing and Recycling of Nonferrous Metals, Lanzhou University of Technology, Lanzhou 730050, China; wukefan1030@163.com (K.W.); yjyan@lut.edu.cn (Y.Y.); zchen@lut.edu.cn (J.C.); 2 School of Materials Science and Engineering, Lanzhou University of Technology, Lanzhou 730050, China; 3 Atlantic China Welding Consumables, Inc., Zigong 643000, China; lixy5011@126.com (X.L.); jy70c6@yeah.net (Y.J.); feifeimy1977@163.com (F.Y.); wangwang0114@126.com (X.J.)

**Keywords:** 2.25Cr-1Mo-0.25V weld metal, low-temperature impact toughness, coarse ferrite, austenite, fracture

## Abstract

The demand for heat-resistant steel has increased owing to its utility in numerous devices that must withstand high steam pressures and high temperatures, such as turbine rotors and blades in ultra-supercritical power plants. It is inevitable to join heat-resistance steel part by welding method, so it is important to maintain the toughness of the weld metals. In this study, the microstructure, low-temperature impact toughness, and fracture surface of as-welded and post-weld heat treatment (PWHT) of 2.25Cr-1Mo-0.25V weld metal were investigated. The microstructures of the as-welded and PWHT specimens are granular bainite and ferrite, respectively. This work revealed the relationship between effective microstructure nearby crack initiation origin and low temperature impact toughness for both the as-welded and PWHT specimens. The evolution of the microstructure and prior austenite was then investigated using confocal laser scanning microscopy (CLSM) to observe the formation of coarse ferrite grain structures. A suggestion for enhancing the low-temperature toughness was provided based on the effect of adjusting Mn content and forming acicular ferrite.

## 1. Introduction

The rapid development of modern, ultra-supercritical power plants has increased the demand for heat resistant steel in numerous devices that operate at high steam pressures and high temperatures such as turbine rotors, blades, and other rotating parts [1]. Heat-resistant steel has a high temperature strength, creep strength, and corrosion resistance. The ferritic heat-resistant 2.25Cr-1Mo-0.25V steel possesses a higher conductivity and a lower coefficient of thermal expansion than that of austenitic heat-resistant steels. Chromium (Cr) predominantly offers oxidation and corrosion resistance to the ferritic heat-resistant 2.25Cr-1Mo-0.25V steel. An increase in the Cr content can enhance the hardenability of heat resistant steel owing to its solid-solution strengthening effect [2,3]. At the same time, molybdenum (Mo) has long been used for heat resistance in order to enhance the creep strength. The Mo can effectively reduce the bainite transformation temperature and delay the grain boundary ferrite transformation, thereby promoting the formation of the bainite microstructure at a wide range of cooling rates [4]. The increase in Mo could stabilize the carbide and further contribute to the high-temperature strength of heat-resistant steel. Vanadium (V) has a strong affinity for other elements such as carbon and nitrogen and is, thus, present in the form of Vanadium Carbide (VC) or Vanadium Nitride (VN) precipitates [5]. VN can inhibit the growth of austenite grain, while the vanadium can increase the strength by precipitation hardening [6]. The ultimate tensile strength of the 2.25Cr-1Mo-0.25V steel at room temperature is higher 70 MPa than that of 2.25Cr-1Mo steel [7,8,9]. Meanwhile, due to the weight reduction and energy conservation of the 2.25Cr-1Mo-0.25V steel, it was quickly brought up to the material standards of ASME (USA), ASTM (USA), and JIS (JPN). Jiang et al. [10,11,12] have investigated the effect of different heat treatments on the microstructure and mechanical properties of 2.25Cr-1Mo-0.25V steels; the results show that appropriate pre-tempering treatment, tempering temperature, and time can enhance the strength and toughness of the steel. Ohtani et al. [13] have investigated the evolution of the microstructure during the creep process. The increase in creep time promotes an increase in the size of the ferrite and the precipitates, thereby resulting in a decrease in the hardness. As V mainly forms complex precipitates with various elements, numerous pieces of research [12,14,15] have focused on investigating the precipitates in the 2.25Cr-1Mo-0.25V steel. After tempering, the majority of precipitates exist in the form of M_23_C_6_ and M_7_C_3_ as tempering decomposes the MC precipitates. Moreover, multiple reports have proposed the addition of various alloys/elements to Cr-Mo-V steel, such as Ti [16,17], B [18], and Re [19,20]. These elements can improve the strength and toughness by refining the grains and forming microstructure, such as acicular ferrite. However, 2.25Cr-1Mo-0.25V steels frequently fail after long-term service [21,22].

Generally, welded joints have three parts including weld zone, heat affected zone (HAZ) and base metal. This study focuses on weld zone of 2.25Cr-1Mo-0.25V welded joints and called it a weld metal. Several challenges exist when the 2.25Cr-1Mo-0.25V heat-resistant weld metal is subjected to the submerged arc welding (SAW) process. As one of the most commonly used fusion welding processes in the industry, SAW can produce high-quality weld metals with a high deposition rate. However, improper SAW parameters may result in slag inclusions, crystal cracking, and hydrogen cracking. Therefore, the correct choice of SAW parameters and the appropriate flux chemical composition are essential to obtaining a high-quality weld metal. However, the presence of oxygen in the flux is unavoidable and can lead to the formation of oxide inclusions in the weld metal, which are likely to accelerate crack initiation in the Charpy impact test. Moreover, diversity in the microstructure of the weld metal was inevitable because the proportion and size of the microstructure are easily influenced by welding process parameters and differences in the chemical composition of the filler metal. In the multi-pass weld metal, different heat inputs and cooling rates [23] generally influence the proportions of the columnar grain zone and reheated regions [24] and the characteristics of the transformation products, such as grain size, impurity or precipitate coarsening of alloying elements [25], then further affect the tensile strength and impact toughness. Furthermore, the welding speed, inter-pass temperature, and post-weld heat treatment (PWHT) can affect the properties of weld metals, particularly the impact toughness. Several studies have reported that a lower impact toughness or unstable toughness can be achieved in the multi-pass weld metals [26,27]. Thus, the 2.25Cr-1Mo-0.25V weld metals with improved impact toughness have important engineering significance for the safe and stable operation of related metal-based equipment.

The strength and toughness are a pair of contradictory unity. Improving strength will result in the reduction of toughness. Post-weld heat treatment (PWHT) is an effective measure of strengthening and toughening, it can also improve fatigue resistance, prolong service life and remove residual stress. In fact, the most beneficial effects of PWHT is improvements of metallurgical structure by tempering and removal of aging effects [28]. Adjusting the process of Quenching and Tempering (QT) can make phase transformations, further achieving maximum toughness and ductility at a specified hardness and strength [29]. There are some alternative methods for PWHT in recent years. For example, temper bead welding (TBW) has the similar effect with PWHT, which attempts to control deposition of the weld layers during weld repairs [30]; Post-weld normalizing heat treatment (PWNT) can eliminate the δ-ferrite in the weld zone and refine the grain structure, which divided PWHT into two steps, first normalizing and then tempering [31].

In order to further enhance the applicability of the heat-resistant 2.25Cr-1Mo-0.25V weld metal, it is critical to understand the relationship between the impact toughness and microstructure of the weld metal. Therefore, this study systematically investigated the low-temperature impact toughness of the heat-resistant 2.25Cr-1Mo-0.25V weld metal manufactured by submerged arc welding and focused on the preferred microstructure of nearby fracture surfaces. The preferred microstructure is characterized by optical microscopy (OM) and scanning electron microscope (SEM). Meanwhile, the novelty of this study is the evolution of those preferred microstructure, which is characterized by confocal laser scanning microscopy (CLSM). According to the analysis and observation of cleavage fracture surface and microstructure, the relationship between the microstructure and low-temperature impact toughness of the as-welded and post-weld heat treatment (PWHT) specimens can be revealed. Based on this relationship, a method for improving the impact toughness was attempted.

## 2. Experimental

### 2.1. Materials and Welding Patameters

**Figure 1:**
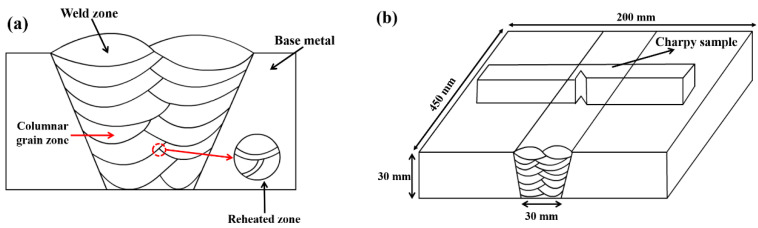
Schematic diagrams of (**a**) multi-layer multi-pass welding welded joint and (**b**) welded joint sampling impact specimen.

In this study, the 2.25Cr-1Mo-0.25V welded joint was obtained by submerged arc welding with 2 beads and 7 layers and the base metal was 2.25Cr-1Mo-0.25V steel. The welding parameters are in accordance with manufacturer data: a pre-heat temperature of 200–250 °C, a voltage of 30 V, a current of 540–550 A, a welding speed of 45 cm/min, a heat input of 301.7 kJ/mm, and an inter-pass temperature of 250 °C. The schematic diagram of multi-layer multi-pass weld is shown in Figure 1a and schematic of welded joint is shown in Figure 1b. Due to the characteristics of multi-layer multi-pass welding, columnar grain zone and reheated zone existed in weld zone. All the experimental materials in this study are taken from weld zone in the joint, not involving heat-affected zones and base materials. The chemical composition of the weld zone is displayed in Table 1 measured by Optical Emission Spectrometer (Spectro lab M9, SPECTRO, Kleve, Germany) after welding.

### 2.2. Thermal Expansion Experiment

**Figure 2:**
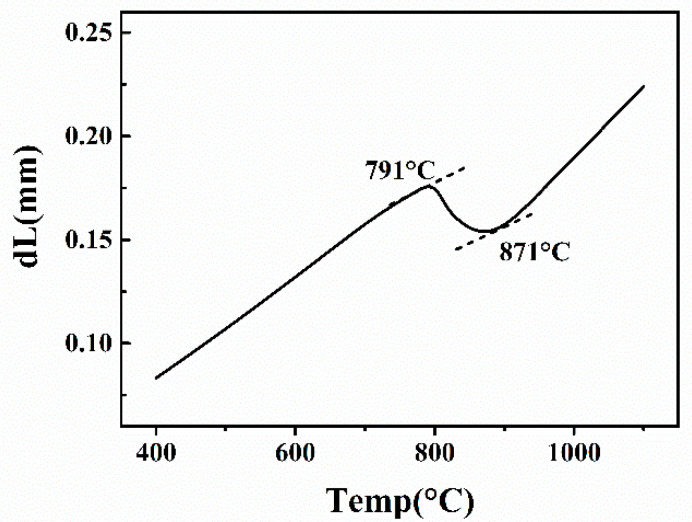
Thermal expansion curve of as-welded specimen.

To ensure favorable mechanical properties of the 2.25Cr-1Mo-0.25V weld metal at high temperature, post weld heat treatment (PWHT) must be carried out, and a tempering heat treatment temperature lower than the austenite transformation start temperature (Ac_1_) are required. Thus, it is necessary to measure the austenite transformation start temperature (Ac_1_) and the austenite transformation end temperature (Ac_3_) by a thermal expansion experiment before the PWHT. The specimen heated up to 1100 °C with a rate of 0.05 °C/s in the thermal expansion experiment. The result of Ac_1_ and Ac_3_ were 791 °C and 871 °C, respectively, as shown in Figure 2. 

### 2.3. Procedure for Post-Weld Heat Treatment (PWHT)

Based on the experimental results of the thermal expansion, PWHT was performed in two stages. First, the post heating treatment was performed; after welding, the plate was immediately heated to 350 °C and maintained for 2 h, then furnace cooled. Second, the principal tempering was performed; the plate was heated to the peak temperature of 705 °C and maintained for 8 h. Finally, it was cooled to 300 °C and then air-cooled to room temperature. 

### 2.4. Charpy Impact Toughness Experiment

The Charpy impact specimens with dimensions of 10 mm × 10 mm × 55 mm (Chinese standard GB/T 229-2007) were machined from the welded joint by an electrical discharge cutting machine (DK7745, HENGTIAN, Jiangsu, China), as shown in Figure 1b. The Charpy V-notch is perpendicular to the welding layer to eliminate the effect of V-notch location (at columnar grain zone or reheated zone). The Charpy impact toughness was measured at −30 °C. The total absorbed energy was identified as the impact toughness. Then, the fracture surfaces were observed by scanning electron microscopy (SEM) (Quanta FEG 450, FEI, Hillsboro, OR, USA). The crack initiation origin on the fracture surface was identified by tracing back the river pattern strips; the fracture surface observation is explained in Section 3.3.

### 2.5. Microstructural Analyses

The metallographic specimens were sampled from the welded joint and then ground, polished, and etched by a 4% Nital solution. The microstructure was observed by optical microscopy (OM) and SEM with energy-dispersive X-ray spectroscopy (EDS).

**Figure 3:**
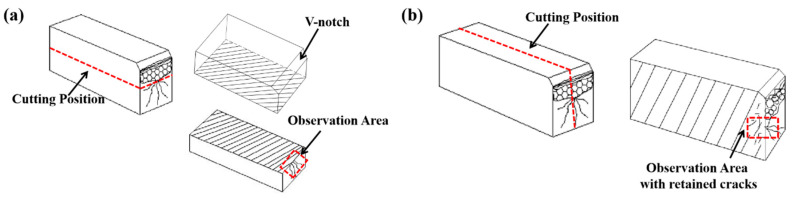
Schematic of metallographic sections from the fractured impact specimens, (**a**) the cutting cross-section parallel to the notch for observation of microstructure nearby fracture initiation, (**b**) the cutting vertical-section perpendicular to the notch for the observation of the retained cracks

To further reveal the relationship between microstructure and the Charpy impact toughness, the present study focused on the effective microstructure at specific areas, such as near the fracture initiation origin and the fracture surface. An electrical discharge cutting machine was used to obtain the observed metallographic sections from the fractured impact specimens. The cutting methods are depicted in Figure 3. The cross-sectional metallographic cutting from the impact specimens is parallel to the notch in order to observe the microstructure at the fracture initiation location, as shown in Figure 3a. The vertical metallographic cutting from the impact specimens is perpendicular to the notch in order to observe the microstructure and retained cracks and to confirm the critical event of crack formation, as shown in Figure 3b. The relationship between microstructure and impact toughness can be explained further by combining two types of metallographic results.

### 2.6. In Situ Observation of Microstructure Evolution

In order to observe the evolution of the microstructure and prior austenite, confocal laser scanning microscope (CLSM) (VL2000DX-SVF18SP, YONEKURA, Tokyo, Japan) experiments were performed. The specimen with dimensions of Ф 7.5 mm × 3 mm was prepared in the as-welded weld zone. It was heated at 1420 °C with a rate of 10 °C/s and held for 240 s to accelerate the evolution of prior austenite and microstructure, then, the cooling rate of 10 °C/s was performed. 

## 3. Experimental Results

### 3.1. The Microstructure of the Weld Metals

**Figure 4:**
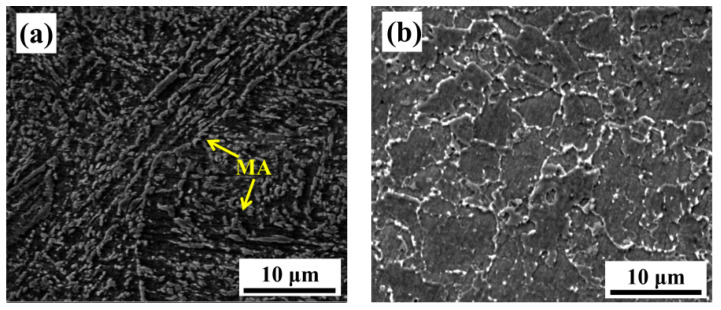

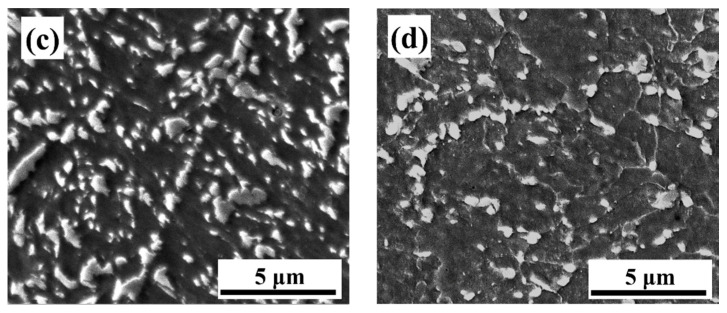
Microstructure of the weld metal for (**a**) as-welded and (**b**) post-weld heat treatment (PWHT) specimens; high magnification images of the weld metal for (**c**) as-welded and (**d**) PWHT specimens.

The microstructure of the as-welded specimen is composed of bainitic ferrite, blocky martensite islands, and retained austenite (M-A constituents) [32], as shown in Figure 4a,c. The slender M-A constituents are distributed in the prior austenite grain boundary, whereas the massive M-A constituents are distributed in the prior austenite grain boundary, respectively. The microstructure of PWHT specimens is shown in Figure 4b. After tempering at the peak temperature of 705 °C and holding for 8 h, the microstructure transformed into ferrite and carbide precipitates, which are distributed in the ferrite grain boundaries, as shown in Figure 4d. The carbide precipitates in the 2.25Cr-1Mo-0.25V weld metal are usually regarded as MC, M_7_C_3_, and M_23_C_6_, where the “M” represents metallic elements such as Cr, Mo, V, and Fe [11,14].

### 3.2. Mechanical Properties of the Weld Metals

**Figure 5:**
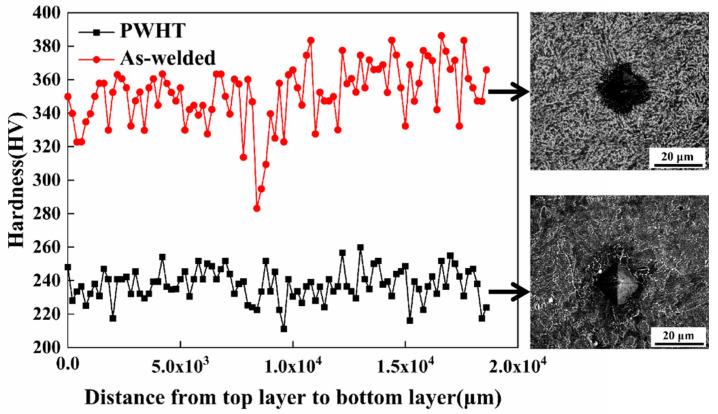
Hardness of the weld metal for as-welded and PWHT specimens.

The mechanical properties tests were performed for the as-welded and PWHT specimens. The tensile results at 20 °C are listed in Table 2. Here the mechanical properties standard of 2.25Cr-1Mo-0.25V steel (base metal) is also presented (ASTM A542D C1 4a). The measured yield strength (YS) and ultimate tensile strength (UTS) of the PWHT specimen are 581 MPa and 695 MPa, respectively, which are lower than that of the as-welded specimen (709 MPa (YS), 965 MPa (UTS)). Both as-welded and PWHT specimens are meeting the strength standard. The hardness results are presented in Figure 5. The hardness of the as-welded and PWHT specimens are within the ranges of 280–380 HV and 210–260 HV, respectively. The SEM images of the hardness indentation and microstructure corresponding to the hardness value are also shown in Figure 5. Although PWHT decreases the yield strength and ultimate tensile strength, the technique still satisfies the strength requirements of the heat-resistant weld metal at 20 °C.

The reduction of the cross-sectional area (Ψ) and Charpy impact toughness are also presented in Table 2. The PWHT process increases the plasticity and results in improved impact toughness from a range of 6–7 J to that of 30–36 J at ‒30 ° C. However, according to the impact energy standard of base metal, the impact toughness of the PWHT specimens is still low and needs to be improved further. The proposed method of improvement in this study is detailed in Section 3.6.

### 3.3. Fracture Surface of the Impact Specimens

**Figure 6:**
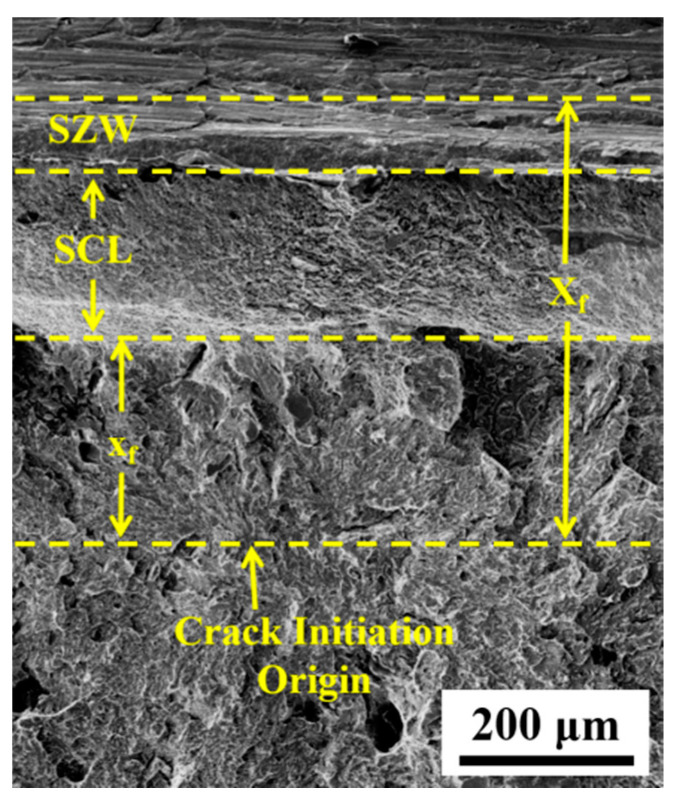
Macro-fracture surface showing the integrating measurements of stretch zone width (SZW), fibrous crack length (SCL), and the x_f_ from PWHT specimen.

The crack initiation origin is identified by tracing back the river pattern strips on the fracture surface. In order to identify the relationship between impact toughness and fracture surface, four parameters of the fracture surface are defined in Refs. [33,34]. These parameters are the stretch zone width (SZW), fibrous crack length (SCL), the distance from the crack initiation site to the fibrous crack (x_f_), and the distance from the crack initiation location to the original notch root (X_f_). The four parameters are related based on the equation X_f_ = SZW + SCL + x_f_, where the sum of SZW and SCL can directly indicate the level of toughness. The corresponding regions are marked in the impact fracture surface of the PWHT specimen with an impact toughness of 32 J, as shown in Figure 6.

**Figure 7:**
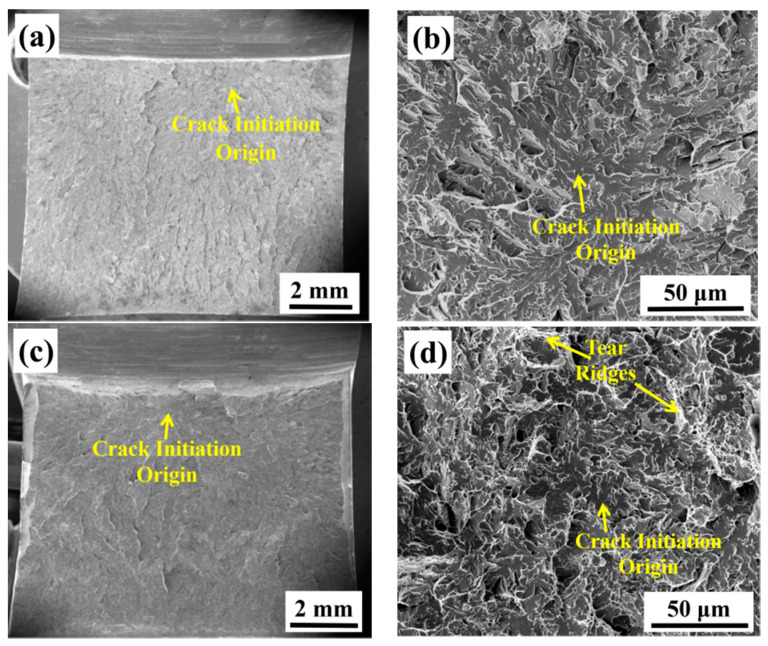
Fracture surface of as-welded specimen (**a,b**) and PWHT specimen (**c,d**).

To further investigate the difference in low-temperature impact toughness between the as-welded and PWHT specimens, the fracture surfaces of the specimens were compared in Figure 7. Figure 7a,b show the fracture surface of the as-welded specimen, while Figure 7c,d show the fracture surface of the PWHT specimen. The fracture surface is composed of the SZW without the typical feature of the nearby V-notch, SCL with dimple feature, and brittle cleavage fracture zone. The fracture initiation origin is tracked by river line features at low magnification, as shown in Figure 7a,c. Figure 7b,d illustrate the magnification of the fracture initiation origin of the as-welded and PWHT specimens, respectively. Quasi-cleavage fracture dominates the fracture surface of the as-welded and PWHT specimens, as shown in Figure 7a,c. More tear ridges with dimples exist in the PWHT fracture surface, which is also one of the factors that render the toughness of the PWHT specimen higher than that of the as-welded specimen, as shown in Figure 7b,d.

The microscopic parameters of the fracture surface can be measured, as shown in Table 3. The sum of the SZW and SCL characterizing the level of impact toughness increased significantly after PWHT. However, the relationship between toughness and fracture surface should be compared one-to-one. The parameters of the fracture surface cannot explain the reason for different toughness values. In fact, the variation in the impact toughness is caused by the dissimilarity between granular bainite for the as-welded specimen and ferrite for the PWHT specimen.

### 3.4. Effective Microstructure for Determining Impact Toughness

Various scholars have postulated the relationship between microstructure and toughness. Pickering et al. [35] indicated that the ductile-brittle transition temperature (DBTT) is represented by the following:

(1) $$\mathrm{DBTT}=f(\mathrm{composition})+g(\mathrm{strength})-11.5\;\times \;{\left(d\right)}^{-1/2}$$

Where f and g are functions of composition and strength, d is the effective grain size. A decreased grain size is beneficial for decreasing the DBTT and improving the low-temperature toughness [12]. Figure 4b shows the microstructure of the 2.25Cr-1Mo-0.25V weld metal after PWHT; the ferrite grain size is only ~10 μm. The microstructure with such fine grain size should not have low impact toughness at low temperature, but PWHT specimens only have ~30 J impact absorbed energy. Therefore, this study focused on the effective microstructure at a specific area, such as the microstructure near the fracture initiation origin and the microstructure with retained cracks near the fracture surface. The retained cracks can exhibit a critical event, which is defined as the most difficult process of crack formation [34]. Thus, the vertical-section must be perpendicular to the fracture surface and located in the region nearly 250 μm from the cleavage fracture initiation origin to observe the retained cracks [36]. The sampling method for the observed metallographic sections is explained in Section 2.5 and Figure 3.

**Figure 8:**
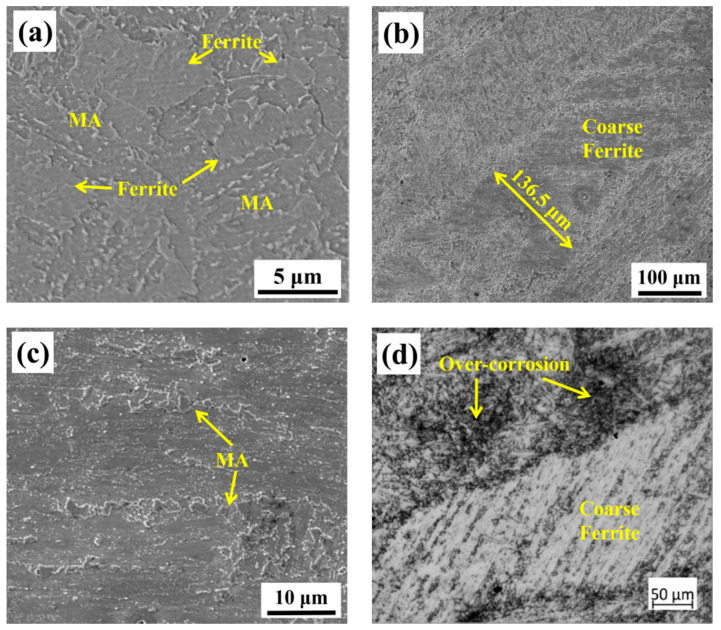
Microstructure of cross-section in Figure 3a for as-welded specimen (**a**), and for PWHT specimen (**b–d**).

**Figure 9:**
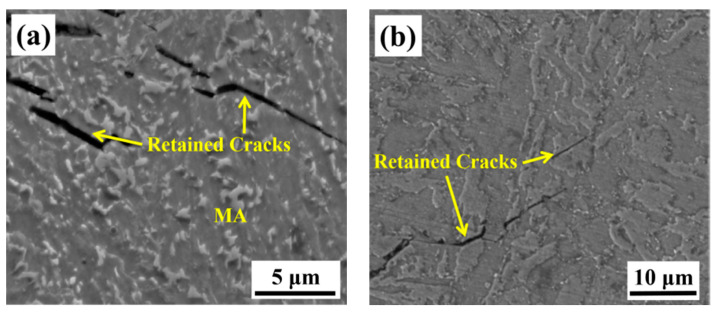
Retained cracks of vertical-section in Figure 3b for as-welded specimen (**a**), and for PWHT specimen (**b**).

The microstructure near the crack initiation origin on the cross-section is shown in Figure 8. The retained cracks on the vertical-section are displayed in Figure 9. Comparing Figure 8a and Figure 4a, it can be seen that the microstructure near the crack initiation origin is different from that in the other zones in the as-welded specimen. The ferrite grains near the crack initiation origin are surrounded by several M-A constituents. From Figure 8a, it can be concluded that the microstructure near the crack initiation has two characteristics. First, the M-A constituents surrounded by ferrite cause stress concentration, which in turn, leads to crack initiation. The second characteristic is the presence of coarse ferrite phases, which do not appear elsewhere. The retained cracks of the as-welded impact specimen shown in Figure 9a and are propagated through bainitic ferrite. This suggests that the critical event in the as-welded specimen is the propagation of a grain-sized crack across the grain/grain boundary into contiguous grains.

The microstructure near the crack initiation origin of the PWHT specimen is different from that of the as-welded specimen. A coarse ferrite appeared near the crack initiation zone, as shown in Figure 8b, which represents the shape of the coarse ferrite with a width larger than 100 μm. It is obvious that the coarse ferrite is located in the columnar grain zone of the weld metal, and the length of the coarse ferrite is consistent with the length of the columnar grain zone. The coarse ferrite in Figure 8c is magnified in Figure 8b. No grain boundaries exist within the coarse ferrite, but some M-A constituents can be found. These M-A constituents were not decomposed by PWHT, which makes coarse ferrite more brittle. In order to eliminate the effect of the corrosion time on the coarse ferrite, the specimen was corroded again for a longer time by 5% Nital solution; the OM image is shown in Figure 8d. Although the ferrite beside the coarse ferrite already exhibited black traces of over-corrosion, no grain boundaries appeared within the coarse ferrite. The retained cracks propagated through the ferrite grain and stopped at the grain boundaries, as shown in Figure 9b. These transgranular retained cracks were hard to break through the ferrite grain boundaries, which means the size of retained cracks grew with grain size. According to the retained cracks in ferrites, the cracks in coarse ferrite will have the same length with coarse ferrites when cracks nucleate inside the coarse ferrite. Therefore, the critical event is confirmed as the propagation of a grain-sized crack across the grain/grain boundary into contiguous grains. This result is consistent with the theory in Equation (1); when the grain size is large, the low-temperature toughness decreases with the increase in the grain size. The coarse ferrite near the crack initiation origin can be regarded as the effective microstructure influencing low-temperature toughness. 

### 3.5. In Situ Observation of Microstructure Evolution

**Figure 10:**
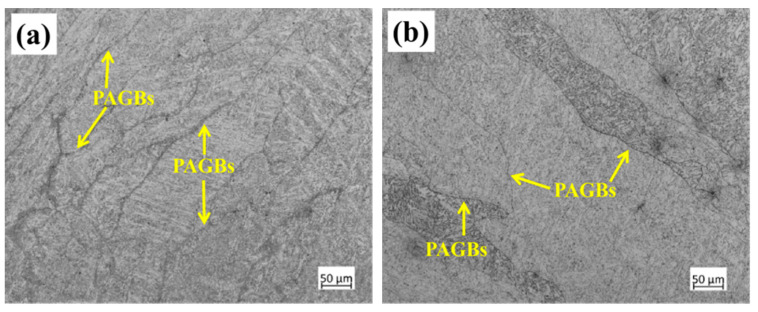
Optical microscopy (OM) images of columnar grain zone of (**a**) as-welded specimen and (**b**) PWHT specimen.

As stated above, the coarse ferrite in the PWHT weld metal induces specimen fracture and leads to lower impact toughness. One of the characteristics of coarse ferrite is that it has the same length as the columnar grain zone. The OM images of the columnar grain zone are depicted in Figure 10. The prior austenite grain boundaries (PAGBs) of the as-welded and PWHT specimens, as shown in Figure 10a,b, maintain their columnar morphology during tempering heat treatment. It can be inferred that the primary columnar austenite formed during the welding process determined the size of the coarse ferrite. 

**Figure 11:**
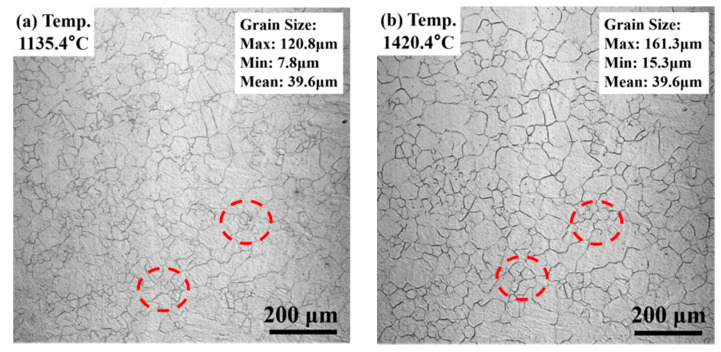

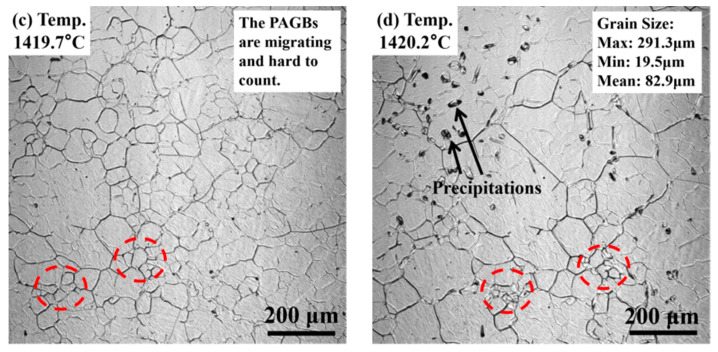
CLSM images of the weld metal for (**a**) heating stage, (**b**) initial stage of temperature holding stage, (**c**) temperature holding stage, (**d**) end stage of temperature holding stage.

**Figure 12:**
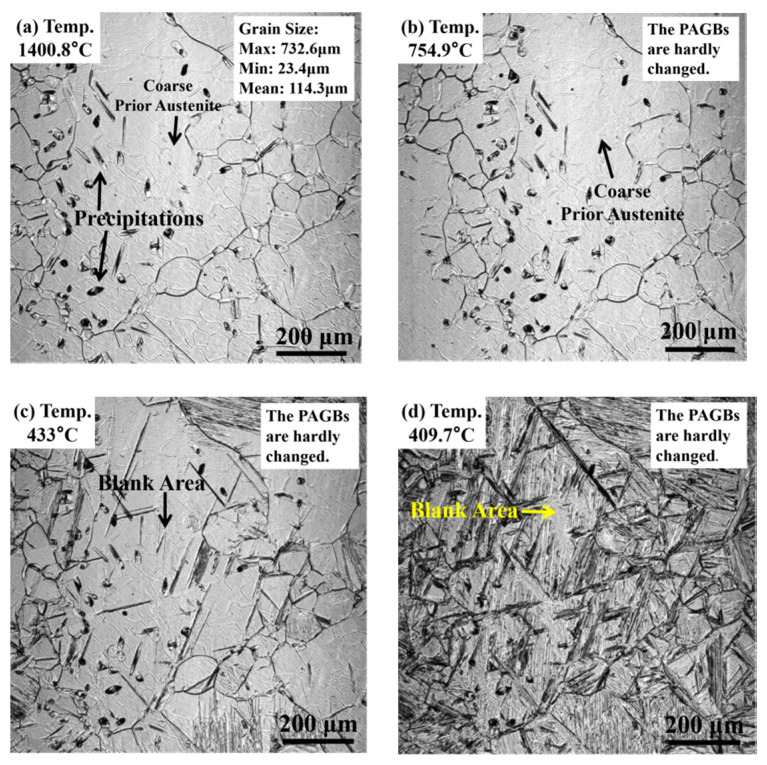
Confocal laser scanning microscopy (CLSM) images of the weld metal for (**a**) initial stage of cooling stage, (**b**) cooling stage at 754 °C, (**c**) cooling stage at 433 °C, (**d**) end stage of cooling stage.

The in-situ observation of the evolution of prior austenite at high temperatures is required to identify the relationship between the prior austenite and the coarse ferrite. The in-situ observation of the evolution of prior austenite by CLSM is shown in Figure 11, Figure 12. Prior austenite first appeared at 1135 °C in the heating stage, as shown in Figure 11a. As the heating temperature was higher than that in Ac_3_ (871 °C), all the prior austenite phases were re-austenitized and homogenized to form fine austenite grains at the early heating stage. The average grain size is 39.6 μm, as shown in Figure 11a. Then, the prior austenite began to grow at the temperature holding stage. The high temperature of 1420 °C accelerated the grain growth rate and made the observation of the grain boundary migration easier, as shown in Figure 11d. The prior austenite grain and statistical results of the grain size at the temperature holding stage are shown in Figure 11b–d. At the initial temperature holding stage, the average grain size only increased 15 μm from the heating stage and reached 55.3 μm. As the temperature is continuously held, the boundary migration phenomena can appear on some fine austenite grain boundaries. At the same time, the fine grain boundaries disappeared and merged into coarse grains. Meanwhile, some austenite maintains its size, as shown in the dotted box. At the end of the temperature holding stage, the largest grain size was 291 μm, while the smallest was 19.5 μm. During the temperature holding stage, not only the prior austenite grain size changed, but some precipitation phases also appeared, as indicated by the arrows in Figure 11d. These precipitates, which were formed at high temperatures, became the inclusions after the cooling process of the weld metal. 

**Figure 13:**
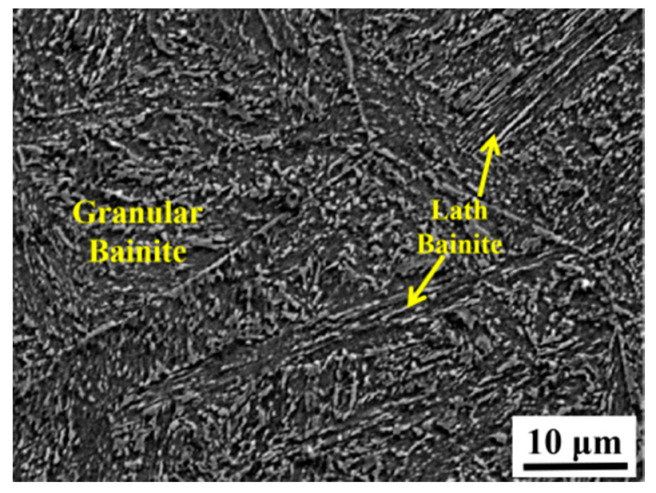
Microstructure of the specimen after CLSM.

The variation of prior austenite during the cooling stage is shown in Figure 12a–d. This study selected another area of observation, which has coarse prior austenite with a columnar-like shape. The grain size of the coarse prior austenite is ~732 μm, while that of the minimum grain is ~ 23.4 μm. Both the coarse prior austenite and the fine prior austenite maintain their size, and no new prior austenite grain boundaries are formed at the cooling stage. When the specimen is cooled to 440 °C, several lath microstructures begin to grow. The lath microstructure nucleates at the prior austenite grain boundaries and inclusions then grows in the inside of the grain. The growth of the lath microstructure reflects the relationship between the coarse ferrite and the prior austenite. The microstructure in the fine prior austenite can occupy the entire area of the grains, while that in the coarse prior austenite will leave a “blank area” with the same growth rate of the lath microstructure, as shown in Figure 12c,d. This “blank area” would form the coarse ferrite and become the effective microstructure of the impact specimen. The microstructure after cooling is shown in Figure 13. The lath microstructure is lath bainite and granular bainite confirmed by SEM image, which is different from the microstructure of the as-welded and PWHT specimens. This type of microstructure was formed by a cooling rate of 10 °C/s, which is located between the air-cooled (in the as-welded specimen) and cooling rate of 55 °C/h (in the PWHT specimen).

### 3.6. Improving the Low-Temperature Toughness of the Weld Metals

**Figure 14:**
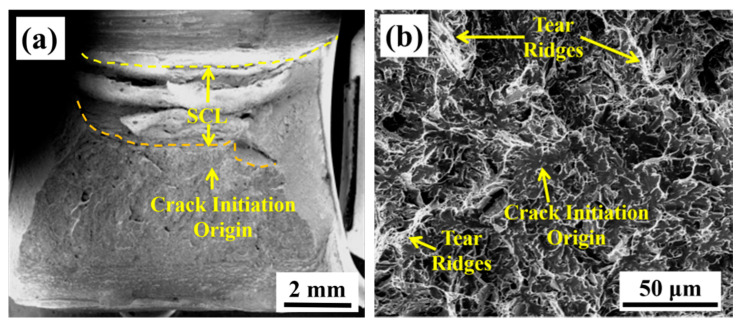
Fracture surface of HM specimen for (**a**) marco, (**b**) crack initiation origin.

**Figure 15:**
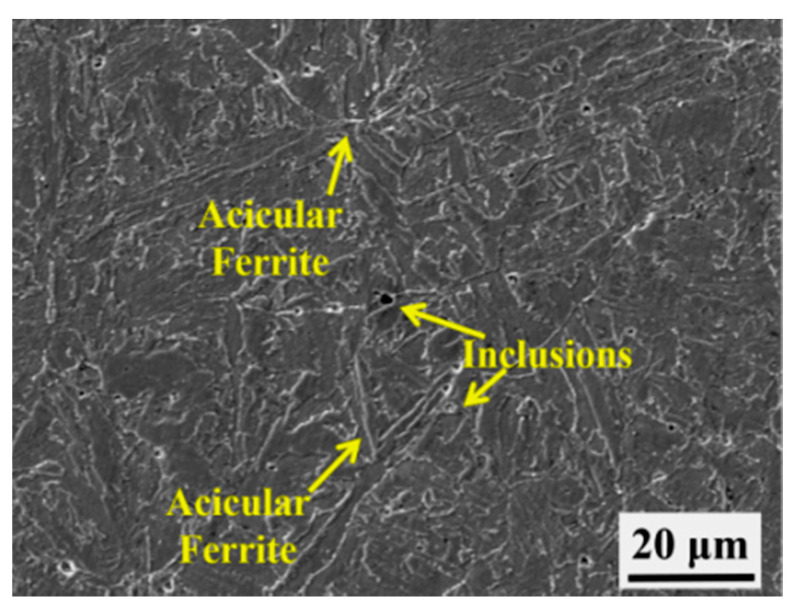
Microstructure of HM specimen.

Based on the above experimental results, improving the low-temperature impact toughness of the weld metals depends on either the decrease of the coarse ferrite or the decrease of the prior austenite grain size. Several studies concentrate on improving impact toughness by changing the elemental composition of the weld metal or the parameters of the PWHT process. This study attempted to improve the toughness by adjusting the manganese (Mn) content from 1.04 to 1.27 wt.% with the other elemental concentrations and PWHT parameters remaining unchanged. The adjusted specimen was recorded as HM. The hardness of the HM specimen is 247 HV. The fracture surface and crack initiation origins and the corresponding low-temperature impact toughness values for the four parameters of the HM specimens are shown in Figure 14 and Table 3, respectively. From Table 3, the impact toughness can be improved from a range of 30–36 J to one of 95–157 J after increasing the manganese concentration. The fibrous crack length (SCL) region with more tear ridges becomes more extensive, as shown in Figure 14. The microstructure of the HM specimen cross-section cutting from the impact fractured specimens is shown in Figure 15. Some acicular ferrite (AF) with inclusions appear in the HM weld metal specimen. The elemental composition of the inclusions was measured via EDS analysis, as shown in Table 4. It can be inferred that the Mn-Si-O inclusions serve as the nucleation sites for the acicular ferrite with the increase of Mn content. 

## 4. Discussion

### 4.1. The Effect of Type of the Microstructure on Mechanical Properties of the Weld Metals

**Figure 16:**
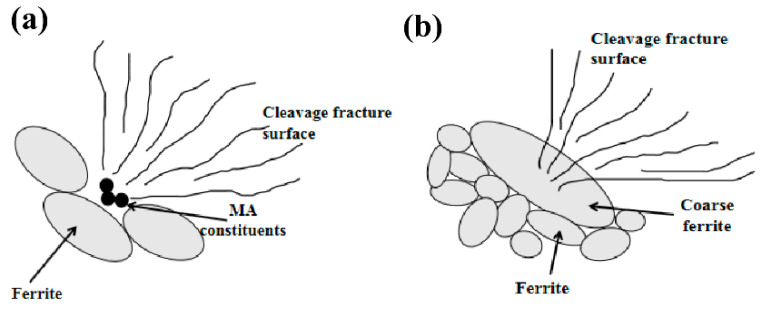
Schematic diagrams showing fracture processes for (**a**) as-welded impact specimen and (**b**) for PWHT impact specimen.

The difference of the mechanical properties for as-welded and PWHT specimens is actually attributed to the difference of the microstructure. The microstructure of as-welded specimen is granular bainite, which is composed of bainite ferrite and M-A constituents. The block M-A constituents are almost considered as strengthening phase. It is the reason why as-welded specimen has higher yield strength, ultimate tensile strength, and hardness, as shown in Table 2. However, from the critical event of an as-welded specimen, as shown in Figure 9a, M-A constituents cannot prevent cracks propagation. The martensite in M-A constituents can also be considered as brittle phase, which is harmful to the impact toughness. But in PWHT specimen, the M-A constituents have been decomposed to carbide precipitation by tempering heat treatment. In as-welded impact specimen, the microstructure nearby crack initiation origin is M-A constituents surrounded by ferrite phases as shown in Figure 8a. Due to stress concentration at M-A constituents, crack can be formed and propagates through the ferrite grain. Schematic diagram of microstructure and impact fracture surface of as-welded specimen is shown in Figure 16a.

The hardness and yield strength of the PWHT specimen with ferrite microstructure are lower than that of as-welded specimen. Thus, lower yield strength leads to lower driving force to cleavage fracture, i.e., Lower σ_yy_=Qσ_y_, where σ_y_ is the normal tensile stress, σ_yy_ is the normal tensile stress ahead of the notch root and Q is stress strengthening coefficient [34]. So, the PWHT specimen fractures at higher applied load, that is, higher impact toughness can be obtained. 

The increase of Mn content leads to the appearance of the acicular ferrite in HM specimen. Since acicular ferrite has characteristics of fine size and high density of high angle grain boundaries, it will twist crack propagation path and absorb more energy during impact test [37,38,39]. The acicular ferrite improves the Charpy impact toughness of the weld metal.

### 4.2. The Effect of Ferrite Grain Size on Impact Toughness of the Weld Metals

Criteria for crack propagation from Griffth [40] and Orowan [41] should be considered to identify the relationship between microstructure and toughness. The formula for cleavage fracture stress σ_f(f)_ is given by:

(2) $$\sigma_{f(f)}=\sqrt{\frac{2E\gamma }{\pi (1-\nu^{2})c}}$$

where E is the Young’s Modulus, γ is the surface energy, ν is the Poisson’s ratio, and c is the half crack length. The equation gives the relationship between the size of the critical event and cleavage fracture. Based on the Griffith formula (Equation (2)), J.H. Chen et al. [42,43,44] put forward three types of critical events for cleavage fracture, which are defined as the most difficult stage of crack formation. The first is crack nucleation as the critical event, the second is the propagation of a second-phase particle-sized crack across the particle/grain boundary into a contiguous matrix as the critical event, and the third is the propagation of a grain-sized crack across the grain/grain boundary into contiguous grains as the critical event. The criterion of the third critical event [44] is

(3) $$\sigma_{\mathrm{yy}}\geq \sigma_{f(f)}$$

where σ_yy_ is the normal tensile stress and σ_f(f)_ is cleavage fracture stress. The relationship between the crack and grain size in this critical event, σ_f(f)_, in Equation (2) can be represented by Equation (4) [44].

(4) $$\sigma_{f(f)}=\sqrt{\frac{2E\gamma }{\pi (1-\nu^{2})D}}$$

where D is the grain size, which is the only microstructural variable determining the cleavage fracture stress. In other words, fine grain size contributes to the increase of impact toughness. From Figure 9b, the critical event of the PWHT impact specimen is the propagation of a grain-sized crack across the grain/grain boundary. Equation (4) is suitable for this situation, and the relationship between grain size and impact toughness is confirmed in the weld metal.

Although the higher impact toughness can be obtained after PWHT as discussed in Section 4.1, the effective microstructure of PWHT impact specimen is that of coarse ferrite near the crack initiation, as shown in Figure 8b. With the critical event and the relationship between grain size and crack, the appearance of the coarse ferrite grain in the PWHT specimen makes the crack readily propagate through the coarse ferrite grain and stop at grain boundaries, so that the low impact toughness of the PWHT specimen cannot satisfy the requirement of toughness. The schematic diagram of the microstructure and impact fracture of the PWHT specimen is shown in Figure 16b.

### 4.3. The Effect of Prior Austenite on Low-Temperature Impact Toughness of the Weld Metals

According to the experimental result of the in-situ observation of the evolution of prior austenite by CLSM, a region of the fine prior austenite in the weld metal can become coarser and reach the same width as the columnar grain zone, whereas the others remain finely sized in the welding process. The coarse prior austenite grain determines the formation of the coarse ferrite in the weld metal. The size of the prior austenite also directly affects the level of impact toughness. The Hall-Petch relationship has a formula (Equation (5)) for the ductile-brittle transition temperature, which reveals the relationship between the grain size and mechanical properties [45].

(5) $$T_{B}=T_{0}-k_{B}\times d^{-\frac{1}{2}}$$

In Equation (5), T_B_ is the ductile-brittle transition temperature, T_0_ is the initial value of T_B_ when d→∞, k_B_ is coefficient, and d is prior austenite size. With the increase of the prior austenite grain size, the ductile-brittle transition temperature is also increased. Thus, the low-temperature toughness is decreased. In recent years, the toughness of high-strength low alloy steel can be improved by refining the prior austenite grain, because the fine prior austenite increases the density of PAGBs and high angle grain boundaries [46,47]. In fact, the refinement of prior austenite results in a finer packet/block size of the microstructure.

As stated above, in the weld metal, a portion of the prior austenite grew into coarse prior austenite in the welding process. The coarse prior austenite affects low-temperature impact toughness in two aspects. First, the over-sized prior austenite directly results in the reduction of toughness due to the Hall-Petch relation. Second, the over-sized prior austenite can form coarse ferrite phases, which become the effective microstructure of crack initiation.

### 4.4. The Effect of Mn Content and Inclusions on the Low-Temperature Impact Toughness of the Weld Metals

The low-temperature impact toughness of the weld metals increases from the 30–36 J range to the 95–157 J range by adjusting the Mn content from 1.04 to 1.27 wt.% due to the formation of Mn-Si-O inclusions and acicular ferrite. The Mn-Si-O inclusion is a kind of complex particle and acts as nucleation site of acicular ferrite [48,49,50,51]. The oxide particles offer cation vacancies and become preferential nucleation site of precipitation of MnS. With the increase of Mn content, Mn-depleted zone increased in matrix, which offer enough nucleation energy to contribute to acicular ferrite nucleation. 

Mn can improve the hardenability, thereby increasing the strength and hardness of the ferrite and austenite in steels [48]. Generally, the increase in Mn content can compensate for the loss of strength resulting from reducing the carbon content. Mn can also significantly reduce the transformation temperature (bainite transformation temperature, Bs, and martensite transformation temperature, Ms), which helps to improve the stability of the overcooled austenite. 

## 5. Conclusions

In the present study, the relationship between low-temperature impact toughness and microstructure in the 2.25Cr-1Mo-0.25V weld metal was investigated. The four main factors are proposed: type of microstructure, grain size, prior austenite, and inclusions. 

The microstructure of the as-welded weld metal and PWHT specimens (705 °C, 8 h) is composed of granular bainite and ferrite, respectively. The low-temperature impact toughness is increased from 6–7 J for the as-welded specimens to 30–36 J for the PWHT specimens (705 °C, 8 h) due to the difference in the microstructure type. The effective microstructures of crack initiation of the as-welded 2.25Cr-1Mo-0.25V weld metal are M-A constituents and ferrite, which caused fracture by stress concentration at the M-A constituents. The effective microstructure of crack initiation of the PWHT 2.25Cr-1Mo-0.25V weld metal is coarse ferrite. Coarse ferrite is more brittle and contributes to crack initiation and propagation. Coarse ferrite phases can be formed in coarse prior austenite grains. 

The impact of toughness can be improved by adjusting the Mn concentration in the 2.25Cr-1Mo-0.25V weld metal. The increase in the Mn content from 1.04 to 1.27 wt.% leads to the formation of Mn-Si-O inclusions and acicular ferrite. 

## Figures and Tables

**Table 1:** Chemical compositions of the weld zone (wt %).

C	Mn	Si + Cr + Mo + Ni + V	S + P
0.094	1.04	3.9	0.0101

**Table 2:** Mechanical properties of the weld zone.

Specimens	YS(MPa)	UTS(MPa)	Ψ(%)	Charpy Impact Energy (J)
As-welded	709	965	64%	6, 6, 7 (−30 °C)
PWHT	581	695	71%	24, 32, 36 (−30 °C)
Standard (BM)	≥ 415	585–760	≥ 45	≥54 (−18 °C)

**Table 3:** Impact toughness of the weld metals.

As-Welded	Impact Toughness (−30 °C) (J)	x_f_ (μm)	SCL (μm)	SZW (μm)	X_f_ (μm)
1	6	188.6	31.1	47.7	267.4
2	6	258	29	31.1	318.1
3	7	185.8	37.3	65.3	288.4
**PWHT**	**Impact toughness (−30 °C) (J)**	**x_f_ (μm)**	**SCL (μm)**	**SZW (μm)**	**X_f_ (μm)**
1	30	587.7	365	117.5	1070.2
2	32	458	432	267.7	1157.7
3	36	504.4	511	286.4	1301.8
**HM(Mn1.27wt%)**	**Impact toughness (−30 °C) (J)**	**x_f_ (μm)**	**SCL (μm)**	**SZW (μm)**	**X_f_ (μm)**
1	95	571	1119.2	340.4	2030.6
2	132	830	2267.3	271.2	3368.5
3	157	629.2	3160.9	219.5	4009.6

**Table 4:** Chemical compositions of inclusions in HM specimen (wt%).

O	Mn	Si	C	Al	Fe	V	Al	Mo
41.73	21.92	16.79	6.11	3.58	4.03	3.92	3.58	1.82
